# Case Report: a giant gastric stromal tumor spontaneously hemorrhaged after freeze-dried rabies vaccine (Vero-cells) injection for human use

**DOI:** 10.3389/fonc.2026.1792937

**Published:** 2026-04-13

**Authors:** Jiayan Shen, Weiwei Wang, Chao Xu, Rubin Ke, Yingshuang Huang

**Affiliations:** 1 Department of Gastroenterology, Hangzhou Linping District Integrated Traditional Chinese and Western Medicine Hospital, Hang Zhou, Zhejiang, China; 2 Department of Gastrointestinal Surgery, Hangzhou Linping District Integrated Traditional Chinese and Western Medicine Hospital, Hang Zhou, Zhejiang, China; 3 Department of Pathology, Hangzhou Linping District Integrated Traditional Chinese and Western Medicine Hospital, Hang Zhou, Zhejiang, China

**Keywords:** adjuvant therapy, freeze-dried rabies vaccine (Vero-cells), gastrointestinal stromal tumors, giant, mechanism, spontaneous hemorrhage

## Abstract

The freeze-dried rabies vaccine (Vero-cells) is generally safe. We present a rare case of a 44-year-old man who developed spontaneous intraperitoneal hemorrhage from a giant gastric gastrointestinal stromal tumor (GIST) shortly after receiving this vaccine. Emergency surgery was performed, and postoperative pathological and immunohistochemical analysis confirmed the diagnosis of a high−risk gastric GIST. The patient declined postoperative adjuvant therapy with imatinib. Follow-up evaluations at one year postoperatively indicated stable disease, and he has remained progression-free for 16 months to date, underscoring the indolent biology of gastric GISTs even in high-risk cases. This case serves as an important reminder for clinicians to consider underlying occult GISTs when evaluating acute abdominal symptoms after vaccination. Through this patient’s journey, we emphasize the significance of performing comprehensive computed tomography (CT) examinations in patients with giant GISTs prior to rabies vaccination, and underscore the crucial need for heightened vigilance and close monitoring following administration of the freeze-dried rabies vaccine (Vero cells). Potential mechanisms that have been hypothesized to explain spontaneous tumor hemorrhage in this context include vaccine−induced inflammatory cytokine surges and hemodynamic changes. Although a rare case of rabies vaccine-associated thrombocytopenia has been reported, evidence for this mechanism remains limited. Further studies are warranted to clarify the relationship between vaccination and tumor rupture.

## Introduction

Gastrointestinal stromal tumors (GISTs) are the most common soft tissue sarcoma that can develop in anywhere in the gastrointestinal tract and are most common in the stomach (60-70%), followed by the small intestine (20-25%), resulting primarily from KIT or PDGFRA activating mutations (1, 2). A global estimate of incidence is 10–15 per million annually and increased substantially over time, typically present in older individuals (≥50 years) (3, 4). The clinical manifestations vary depending on location and size of GISTs, which present with gastrointestinal symptoms such as nausea, vomiting, abdominal pain, abdominal distension, gastrointestinal bleeding, etc. Some patients also have acute abdomen caused by tumor rupture, gastrointestinal obstruction and peritonitis, which requires urgent medical attention and treatment. Generally, small GISTs (≤2cm) grow slowly and are asymptomatic (5). They are often incidentally detected during physical examination and may remain stable for a long time or even throughout life. According to current guidelines, GISTs smaller than 2 cm may be suitable for surveillance in certain circumstances, although this is not an absolute rule and management should be individualized. If GISTs gradually grow up, the growth rate will gradually accelerate, and the degree of malignancy will increase (6). As a result, in cases where GISTs are large and non-metastatic, surgery is the criterion standard treatment approach. Emergency surgical intervention is indicated in cases of complete intestinal obstruction, gastrointestinal perforation, severe gastrointestinal hemorrhage unresponsive to conservative management, or acute intra-abdominal bleeding resulting from spontaneous tumor rupture. The decision to administer adjuvant therapy following surgical resection depends on several clinicopathological factors, including risk stratification, estimated recurrence risk, presence of tumor rupture, and molecular genetic profile (7). Adjuvant therapy has demonstrated clinical benefit predominantly in patients with KIT-mutated GISTs (8). Imatinib is currently recommended as adjuvant treatment after complete resection of localized GISTs and also serves as first-line systemic therapy for unresectable, recurrent, or metastatic disease (9, 10). Sunitinib is approved as second-line therapy, followed by regorafenib as a third-line option, both of which have shown efficacy in managing advanced GISTs (11).

Vaccination is an important prevention and control measure for rabies. At present, the human rabies vaccines used in China mainly include three types, produced using Vero cells, primary hamster kidney cells, or human diploid cells (12). Vero cells have the advantages of rapid reproduction, easy control of exogenous factors after establishment of the cell bank, and the ability to be produced on a large scale using microcarrier bioreactors. Therefore, freeze-dried human rabies vaccine (Vero cells) occupies the main market and has been proven to have good safety and immunogenicity (13).

We presented a case of giant gastric stromal tumor with spontaneously intraperitoneal hemorrhage following injection of freeze-dried rabies vaccine (Vero-cells) for human use. Preoperative radiography suggested a huge mass in the abdominal cavity, and the diagnosis of giant gastric stromal tumor was first considered. Massive hemorrhage in abdominal cavity caused by rupture of giant gastric stromal tumor was confirmed during the operation. The diagnosis of gastric stromal tumor was confirmed by postoperative pathology and immunohistochemistry. Therefore, although the freeze-dried rabies vaccine (Vero-cells) has shown favorable safety and immunogenicity, clinicians should maintain heightened vigilance for rare but potentially severe post-vaccination events. Specifically, individuals with giant GISTs may face an under-recognized risk of intratumoral hemorrhage after immunization; prompt imaging examinations and close monitoring after vaccination are warranted in such cases.

## Case presentation

**Figure 1:**
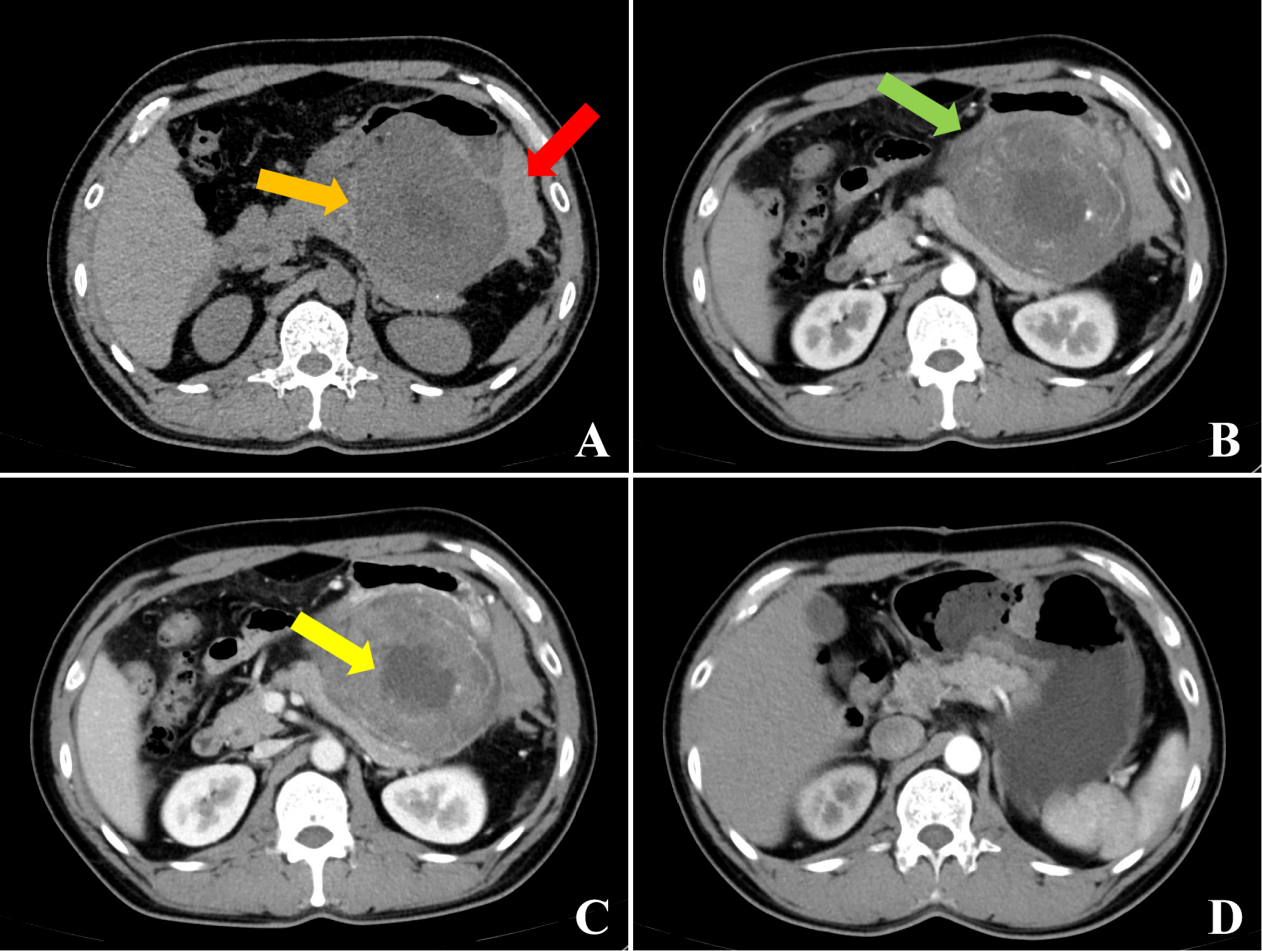
Relevant imaging data during the course of the disease. Abdominal CT scan revealed a space-occupying lesion in the upper left abdomen [**(A)**; orange arrow] and suspicious left upper abdominal hemorrhage [**(A)**; red arrow]; contrast-enhanced CT scan showed a large mass in the left upper abdominal cavity, with uneven density and irregular shape [**(B)** arterial phase; green arrow], and intratumoral low density were indicative of associated hemorrhage and necrosis [**(C)** portal venous phase; yellow arrow]. Contrast-enhanced CT at 1-year postoperation indicated no recurrence in the surgical area [**(D)** arterial phase].

**Figure 2:**
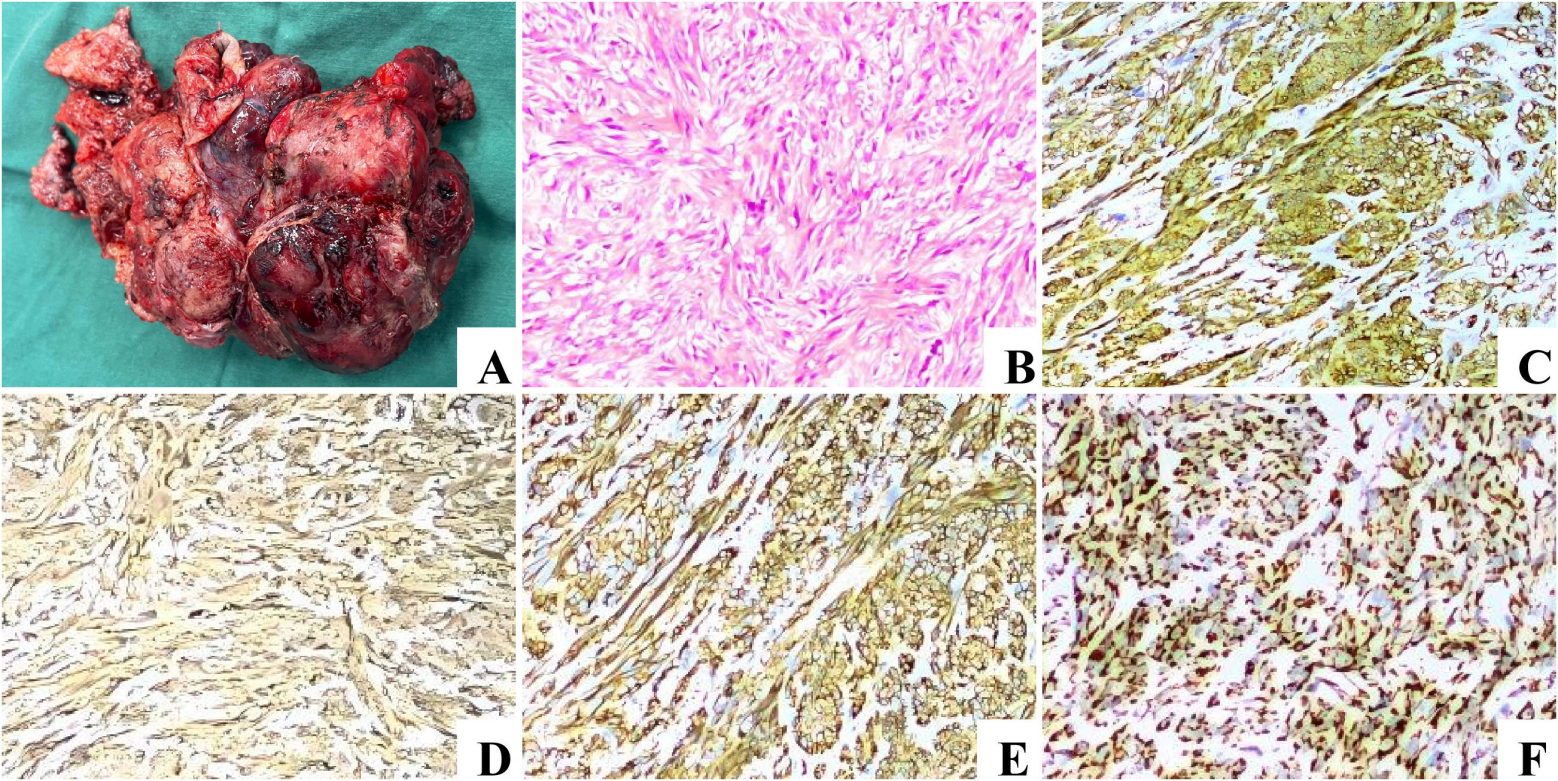
The postoperative pathology. Postoperative specimen shows tumor size of approximately 12 cm × 10 cm × 8.5cm **(A)**. Histologic result showing spindle cell tumor with cell dysplasia [**(B)**: hematoxylin and eosin stain, object lens × 10]. Immunohistochemistry reveals that the tumor is positive for CD117 [**(C)**: immunohistochemical, object lens × 10], CD34 [**(D)**: immunohistochemical, object lens × 10], DOG-1 [**(E)**: immunohistochemical, object lens × 10] and SDHB [**(F)**: immunohistochemical, object lens × 10].

**Figure 3:**
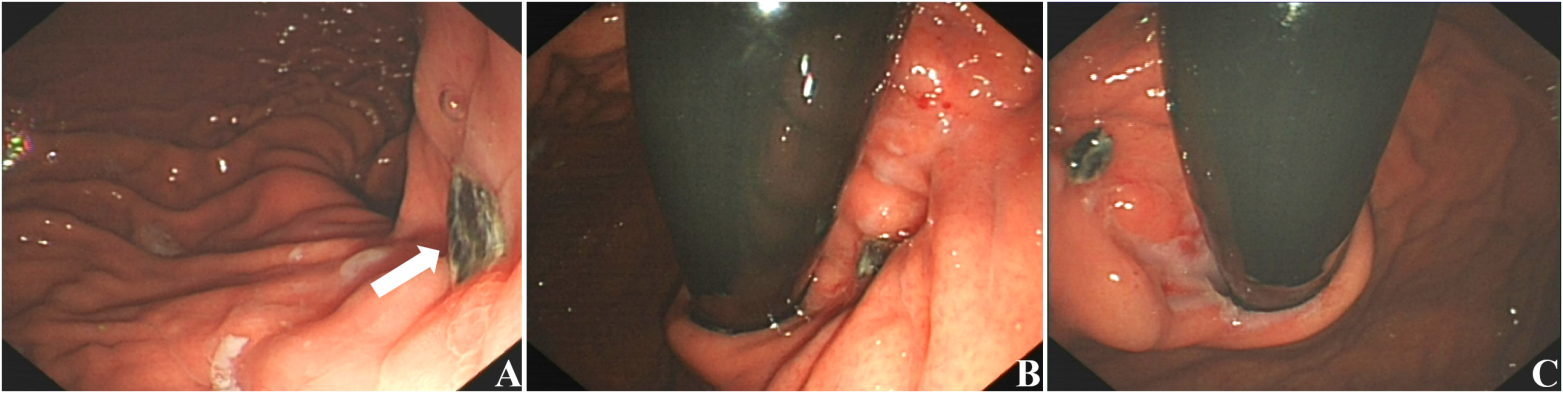
Gastroscopy at 1-year postoperation. The gastroscopy results indicated that there was a scar near the cardia of the fundus, with a remaining black suture line [**(A)**; white arrow], and the cardia mucosa was congested. **(A–C)** show the gastric body’s lesser curvature (en face view), anterior wall (retroflexed view), and posterior wall (retroflexed view), respectively.

**Figure 4:**
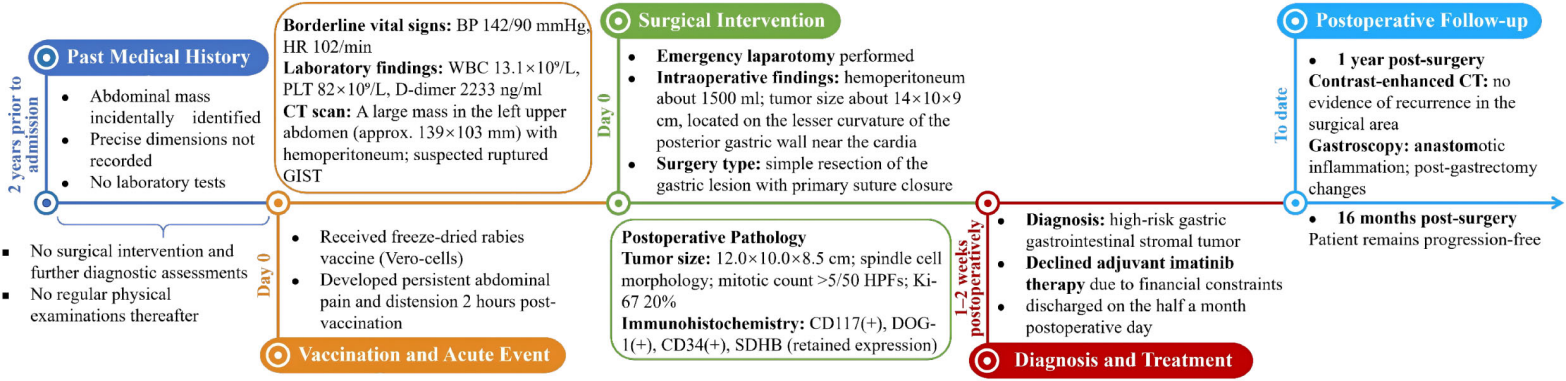
Timeline of clinical events from initial mass identification to postoperative follow-up. Approximately two years prior to presentation, an abdominal mass was incidentally identified but the patient declined further evaluation. On day 0, the patient received the freeze-dried rabies vaccine (Vero-cells) and developed abdominal pain two hours later. Emergency laboratory tests revealed thrombocytopenia (PLT 82×10^9^/L) and elevated D-dimer (2233 ng/ml). Contrast-enhanced CT showed a large mass (139×103 mm) with hemoperitoneum. The patient underwent emergency laparotomy with simple resection of the gastric lesion and primary suture closure. Pathological examination confirmed a high-risk gastric GIST. The patient declined adjuvant imatinib. At one-year follow-up, contrast-enhanced CT and gastroscopy showed no evidence of recurrence. The patient remained progression-free at 16 months postoperatively. BP, blood pressure; CT, computed tomography; GIST, gastrointestinal stromal tumor; HPF, high-power field; HR, heart rate. PLT, platelet; WBC, white blood cell.

We present the case of a 44-year-old male who presented to the emergency department with mild and tolerable abdominal pain and distension 2 hours after receiving freeze-dried rabies vaccine (Vero-cells) for human use. The abdominal pain was a persistent distending pain around the umbilical area, without radiating pain, nausea, vomiting or diarrhea. The patient presented with borderline vital signs, including a blood pressure of 142/90 mmHg and a heart rate of approximately 102/min; all other findings were unremarkable. Two years ago, an abdominal mass was identified during a routine physical examination, but its precise dimensions are not recalled. However, the patient declined the subsequently recommended surgical intervention and further diagnostic assessments. Consequently, no laboratory tests were performed at that time; prior medical records were unavailable for review, and the patient did not undergo regular physical examinations in the years that followed. No previous history of smoking or alcohol consumption. In addition, there was no abnormal health information regarding first-degree relatives. Clinical examination revealed tenderness throughout the abdomen on deep palpation, predominantly around the umbilicus, suspicious rebound tenderness, whereas abdominal rigidity, and guarding were absent. After admission, relevant examinations were completed, and the white blood cell (WBC) was 13.1×10^9^/L, hemoglobin (HB) was 124 g/L, platelet (PLT) was 82×10^9^/L, C-reactive protein (CRP) was 12.1 mg/L and D dimer was 2233ng/ml. CEA, AFP, CA19–9, and CA72–4 indicators returned normal as well as coagulation function. Abdominal CT (**Figure 1A**) revealed a space-occupying lesion in the upper left abdomen, abdominal and pelvic effusion, suspicious left upper abdominal hemorrhage. Therefore, enhanced CT scan of the abdomen and pelvis was further performed and the result revealed a large mass in the left upper abdominal cavity, with uneven density and irregular shape and a maximum cross-sectional size of approximately 139 mm × 103 mm (**Figure 1B**), no abnormal enlarged lymph nodes were found. The origin of the mass may be from the stomach, and the possibility of stromal tumor with rupture and hemorrhage was considered (**Figure 1C**). Unfortunately, we were not able to perform further examination, such as CT-guided puncture of the mass or gastroscopy, to confirm the diagnosis, given the patient’s concern for the complications of intra-abdominal bleeding. The patient eventually underwent emergency laparotomy with simple resection of the gastric lesion and primary suture closure under general anesthesia. Intraoperative exploration: Exploration of the abdominal cavity revealed hemoperitoneum of about 1500ml. Preliminary exploration revealed an irregular mass about 14cm × 10cm × 9cm near the cardia on the small curvature of the posterior wall of the stomach, which adhered to the liver, pancreas and duodenum. No abnormality was found in the visible area of other abdominal organs. After excision and separation of omentum and blood vessels surrounding the mass, the tumor was further exposed and was completely removed irregularly about 1cm beyond the edge of the mass. Further exploration revealed that the tumor arose from the lesser curvature of the posterior gastric wall, consistent with an exophytic gastric stromal tumor. The completely resected tumor was firm in consistency and predominantly dark red and grayish-white in color. It exhibited expansile growth with a nodular or lobulated external surface. The postoperative period was uncomplicated and the patient was discharged on the half a month postoperative day. Postoperative histopathological examination (**Figure 2**) revealed a gastric spindle cell neoplasm, measuring approximately 12.0 × 10.0 × 8.5 cm, which was closely associated with the muscularis mucosa. The tumor exhibited cytological atypia, a mitotic count of >5/50 high-power fields (HPFs), and focal hemorrhagic necrosis. Immunohistochemically, the tumor cells were positive for CD117, DOG-1, CD34, CD31, and SDHB (retained expression), with a Ki-67 proliferation index of approximately 20%. Staining was negative for cytokeratin (CK), desmin, S-100, and smooth muscle actin (SMA). Based on these morphological and immunohistochemical features, the tumor was diagnosed as a high-risk gastric stromal tumor. He declined the postoperative imatinib treatment due to economic reasons. One year after the surgery, an abdominal enhanced CT examination was conducted, which indicated no recurrence in the surgical area (**Figure 1D**). A gastroscopy was also performed, suggesting anastomotic inflammation and changes after partial gastrectomy (**Figure 3**). The patient has remained progression-free for 16 months to date (**Figure 4**).

## Discussion

Gastric GISTs pursue a more indolent biology compared with intestinal or rectal GISTs, and long-term outcome is chiefly determined by complete R0 resection, the mitotic rate, tumor size and anatomic location (1). Although the present patient met high-risk criteria and declined adjuvant imatinib, he remains recurrence-free, underscoring the favourable biology of gastric primaries even when optimal systemic therapy is withheld. However, studies have demonstrated that adjuvant treatment with imatinib can achieve a relatively higher overall survival rate in cases of ruptured GISTs (14); therefore, long-term follow-up and monitoring of the patient are still required.

The freeze-dried rabies vaccine (Vero-cells) for human use has been proved good safety and immunogenicity under both 5-dose and 4-dose immunization procedures (13). The local reactions primarily included pain, itching, swelling, and redness at the injection site, while systemic reactions were mainly fever, fatigue, headache, and myalgia. Most adverse events were of mild severity. It is important to note that adverse reactions to vaccines, particularly those occurring within hours of administration, are often attributable to hypersensitivity responses triggered by non-antigen components rather than the inactivated virus itself (15). The freeze-dried rabies vaccine (Vero-cells) contains not only inactivated rabies virus but also adjuvants, stabilizers, and trace amounts of antibiotics used during manufacturing. These excipients are generally considered safe for the majority of recipients; however, in susceptible individuals, they may provoke an immediate inflammatory response through activation of innate immune pathways. Aluminum-based adjuvants, in particular, have been extensively studied for their immunomodulatory effects. Recent evidence demonstrates that aluminum adjuvants can induce the production of pro-inflammatory cytokines such as IL-1β, IL-6, and TNF-α in human macrophages and other cell types (16). Nies et al. (17) showed that exposure to aluminum-based adjuvants (Alhydrogel and Adju-Phos) decreased cell viability and increased reactive oxygen species formation, accompanied by enhanced secretion of TNF-α and IL-6 from human macrophages. These findings are consistent with the well-established role of aluminum adjuvants in activating the NLRP3 inflammasome pathway, leading to IL-1β release and subsequent inflammatory cascades (18, 19). Such cytokine surges, even if transient, could theoretically alter vascular permeability or exert hemodynamic effects on pre-existing abnormal tumor vasculature, particularly in highly vascularized tumors like GISTs.

Firstly, vaccination and acute viral infection may cause a transient surge of inflammatory cytokines, which may have an “impact effect” on the shear stress of the tumor wall, thereby leading to tumor rupture or bleeding. In 2023, Yu et al. (20) used PIKA as an adjuvant to enhance rabies vaccination. They found that the levels of IL-1β, IL-6, CCL-2 and TNF-α at the injection site increased. Smreczak et al. (21) confirmed in a rabies virus (RABV) infected murine model that the virus group upregulated the expression of IL-6 and TNF-α in the spinal cord and serum. Moreover, in mice inoculated with RABV, the use of TNF-α and IL-6 inhibitors extended mice survival. Furthermore, the acute systemic inflammation triggered by IL-6 and TNF-α may cause hemodynamic changes (22), thereby exerting pressure on the tumor wall and leading to tumor rupture or bleeding. At present, there are no epidemiological or mechanistic reports on spontaneous rupture and bleeding of gastric stromal tumors after rabies vaccine administration. The proposed “vaccine - vascular wall stress - bleeding” conceptual model still requires more clinical validation.

Secondly, GISTs especially giant GISTs with a diameter greater than 10 cm, have a very high probability of spontaneous tumor rupture and hemorrhage. In the study by Joensuu et al., among 358 patients with confirmed localised GIST, 73 (20%) had rupture reported (14). Moreover, the risk of the tumor rupturing and bleeding may also increase when there is a decrease in platelets or a temporary increase in fibrinolysis. This concern is supported by rare but documented evidence of vaccine-associated thrombocytopenia. Bansal et al. (23) reported a case of severe immune thrombocytopenia (ITP) induced by a purified Vero cell culture rabies vaccine. A 38-year-old male developed generalized petechiae and oral bleeds within 12 hours of receiving his first post-exposure prophylaxis dose. His platelet count dropped dramatically to 0.12×10^9^/L, while coagulation times remained normal. The bone marrow examination was consistent with peripheral platelet destruction, confirming the diagnosis of ITP. The patient showed poor initial response to corticosteroids but eventually recovered after treatment with intravenous immunoglobulin and eltrombopag. This case highlights that although extremely rare, anti-rabies vaccines can trigger an immune-mediated destruction of platelets, potentially contributing to a bleeding diathesis.

Last but not least, patients with giant GISTs may represent a potentially vulnerable population requiring clinical attention. As we reported, the patient was incidentally found to have an abdominal mass two years ago, but no further investigation or intervention was pursued at that time. He remained asymptomatic for the subsequent two years. However, following the administration of a freeze-dried rabies vaccine (Vero-cells) on this occasion, he developed acute abdominal pain and distension, which ultimately led to surgical intervention. Postoperative histopathological and immunohistochemical analyses confirmed the diagnosis of a gastric stromal tumor. Accordingly, for patients with known giant GISTs, clinicians may consider baseline CT assessment prior to rabies vaccination on a case-by-case basis, and post-vaccination monitoring for blood pressure, abdominal symptoms, and platelet counts may be prudent.

In light of these findings, the temporal association between rabies vaccination and intratumoral hemorrhage in this case warrants exploration of potential underlying mechanisms, while acknowledging the limitations of current evidence. Notably, the temporal proximity between vaccination and tumor rupture in this case raises a complex question regarding causality. Although a systemic post-vaccination inflammatory response could theoretically influence tumor integrity, there is no established evidence to support a direct causal role. Given the absence of evidence linking rabies vaccination to tumor rupture and the high baseline risk of spontaneous hemorrhage in giant GISTs, a coincidental occurrence appears substantially more likely than a causal relationship. This case highlights the difficulty in distinguishing between causation and coincidence in rare clinical events, and underscores the need for further research to clarify the potential risks of vaccination in patients with occult high-risk tumors.

## Conclusion

In conclusion, by reviewing the journey of our patient and literature research, the Vero-cell vaccine is generally safe. This case presents a rare instance of intratumoral hemorrhage occurring shortly after vaccination in a patient with an incidentally discovered giant gastric GIST. Given the high baseline risk of spontaneous rupture in tumors larger than 10 cm and the absence of established evidence linking rabies vaccination to vascular damage, a causal relationship cannot be inferred. The temporal association observed in this case should be interpreted with caution. Nevertheless, clinicians are advised to conduct a comprehensive evaluation of the patient’s medical history prior to vaccination. For high-risk individuals with giant GISTs (>10 cm), a pre-vaccination CT scan should be considered, when feasible, to evaluate tumor stability and hemorrhage risk. Following administration of the freeze-dried rabies vaccine (Vero cells), individuals with a known history of GIST may benefit from close monitoring for signs and symptoms suggestive of intratumoral hemorrhage. However, the mechanism underlying hemorrhage occurring after rabies vaccination in GISTs, if any, remains elusive. Subsequently, the proposed mechanisms warrant further investigation through animal models, and merit validation in multi-center case-control or cohort studies.

## Data Availability

The original contributions presented in the study are included in the article/supplementary material. Further inquiries can be directed to the corresponding author.
